# The efficacy and safety of several interventions of corticosteroids for CRSwNP patients after endoscope sinus surgery: A protocol for systematic review and network meta-analysis

**DOI:** 10.1097/MD.0000000000031831

**Published:** 2022-11-18

**Authors:** Lan Zhang, Baohua Zhu, Rong Zhang, Huixia Liu, Peishan Li, Jie Liao, Hanchao Shen, Li Tian

**Affiliations:** a Clinical Medicine College, Chengdu University of Traditional Chinese medicine, Chengdu, Sichuan Province, P. R. China; b Department of Otorhinolaryngology, Affiliated Hospital of Chengdu University of Traditional Chinese medicine, Chengdu, Sichuan Province, P. R. China.

**Keywords:** chronic rhinosinusitis with nasal polyps (CRSwNP), corticosteroids, endoscopic sinus surgery (ESS), network meta-analysis

## Abstract

Corticosteroid has been a mainstay of chronic rhinosinusitis with nasal polyps (CRSwNP) medical therapy. While endoscopic sinus surgery (ESS) will be performed when patients had failed to respond to maximal medical therapy. Many studies shown that several corticosteroids of interventions (e.g., nasal spray, oral, atomization/nebulization, nasal irrigation, direct infiltration, and steroid-eluting stent, etc) have each demonstrated significant efficacy compared with placebo or no corticosteroids intervention except intranasal corticosteroids for the treatment of CRSwNP after ESS. The aim of this systematic review and network meta-analysis is to answer the following question: which 1 is the best corticosteroid of intervention for CRSwNP patients after ESS?

**Methods:**

A systematic review will be conducted to identify studies involving randomized controlled trials which compared several different interventions of corticosteroids (e.g., nasal spray, oral, atomization/nebulization, nasal irrigation, direct infiltration, steroid-eluting stent, etc) for CRSwNP patients after ESS. The primary outcomes are efficacy (e.g., effective rate or cure rate), visual analogic scale of symptom severity, Lund-Kennedy endoscopic score, adverse events, and so on. We will comprehensively search PubMed, Embase, Cochrane Library, ClinicalTrials.gov, Web of Science, Chinese National Knowledge Infrastructure, Wangfang and VIP journal database from inception to July, 2022. For studies which meet our inclusion criteria, 2 reviewers will extract data independently and assess the quality of literature using a revision of version 2 of the Cochrane risk of bias tool (RoB 2.0). A random effects model will be used for all pairwise meta-analyses (with a 95% confidence interval). Network meta-analyses will be conducted to generate estimates of comparative effectiveness of each intervention class and rankings of their effectiveness.

**Results:**

The results of this study expect to provide a high-quality, evidence-based recommendation on which 1 is the best corticosteroid of intervention for CRSwNP patients after ESS?

**Discussion:**

This study will provide evidence regarding the comparability of several interventions of corticosteroids for CRSwNP patients after ESS. Also, the data generated from this review will provide health-care providers with a clear evidence synthesis of CRSwNP patients after ESS management strategies. These data will be incorporated into the development of a patient decision aid to assist patients and clinicians in making a preference-based decision when faced with a CRSwNP patients after ESS as well.

## 1. Introduction

As a subgroup of chronic rhinosinusitis, the clinical diagnosis of chronic rhinosinusitis with nasal polyps (CRSwNP) is made on the basis of the presence of sinonasal symptoms (anterior/posterior rhinorrhea, nasal congestion, hyposmia and/or facial pressure or pain) for greater than 12 weeks duration and the visualization of polyps in nasal cavity.^[1–3]^ Nasal polyps are benign inflammatory masses, arising from the mucosa of the nose and paranasal sinuses. CRSwNP affects 1% to 4% of the population, while the etiology and mechanism of CRSwNP are yet to be fully elucidated. CRSwNP will increase heavily direct and indirect economic burdens on patients and society. In the USA, total annual direct health care costs among patients with CRSwNP were $11,507.^[4]^ While in Europe, it was reported that direct costs for CRSwNP patients were €1501 per patient/year, with indirect costs of €5659 per patient/year.^[5]^

While treatment options for patients with CRSwNP remain limited. CRSwNP patients who have failed medical management (such as topical/systemic corticosteroids, antibiotics, nasal saline irrigations, etc) may be eligible for sinus surgery.^[6,7]^ Endoscopic sinus surgery (ESS) is one of the most frequently performed surgical procedures for CRSwNP patients in otolaryngology department, with more than 2,50,000 cases annually in the US.^[8,9]^ Despite CRSwNP patients with maximal medical therapy and even performing ESS, the rate of post-treatment symptom recurrence or need for revision surgery has been estimated to be as high as 14% to 60%.^[10–13]^ Meanwhile, there will be an increasing risk of systemic adverse reactions (e.g., osteoporosis, bone fracture, infections, and gastro-intestinal bleeding, the inhibition of the hypothalamic-pituitary adrenal axis [HPA-axis]) in CRSwNP patients with long-term or repeated using corticosteroids after ESS.

Clinically, CRSwNP with comorbid asthma is associated with more severe sinonasal symptoms and worse quality of life, and it is more difficult to treat both medically and surgically also.^[14]^ A study pointed out that CRSwNP is more commonly associated with several asthma (57.1–62% of patients) than mild asthma (38–42.9% of patients).^[15]^ Several studies shown that patients with CRSwNP and comorbid asthma/allergic rhinitis have higher rates of corticosteroids dependence compared with patients with chronic rhinosinusitis without nasal polyps. Another study shown that patients with CRS with comorbid asthma and nasal polyposis were 6 times more likely to require revision surgery than those with CRS alone.^[16]^ A long-term cohort study pointed out 78.9% of CRSwNP patients who received primary ESS patients would be subjected to recurrence in over a 12-years period.^[17]^ While, Vlaminck S, et al reported that recurrence was observed in 62% of CRSwNP patients in 10 years follow-up.^[18]^

Clinically, Corticosteroids administrations is an important part of the management of CRSwNP after ESS. It can prevent nasal polyps’ recurrence after ESS by decreasing mucosal edema, reducing the eosinophil infiltration, inhibiting the secretion of chemotactic cytokines, and so on. While the types of interventions of corticosteroids after ESS play a vital role in postoperative recovery of CRSwNP patients. Many studies demonstrated that multimodal interventions of corticosteroids (e.g., nasal spray, oral, atomization/nebulization, nasal irrigation, direct infiltration, steroid-eluting stent, etc) have each significant efficacy compared with placebo or no steroids intervention for CRSwNP patients after ESS.^[19–29]^ Despite the growing corticosteroids treatment options for CRSwNP patients after ESS, there is no comprehensive comparison to evaluate the efficacy and safety of all interventions, also there is no consensus on which 1 is the best corticosteroid of intervention for CRSwNP patients after ESS. Therefore, we aimed to systematically review all literature and comprehensively compare and rank the efficacy of various types of interventions of corticosteroids with a network meta-analysis for CRSwNP patients after ESS.

## 2. Methods

We adhere to the Preferred Reporting Items for Systematic Review and Meta-analysis Protocols (PRISMA-P) in this protocol.^[30]^ Similarly, to results of this study will be reported in line with the PRISMA extension for network meta-analyses.^[31]^ This study is registered in the PROSPERO database, and any amendments to this protocol will be indicated there.

### 2.1. Eligibility criteria

Detailed eligibility criteria have been developed following the Population, Intervention, Comparator, Outcomes, and Study Design (PICOS) format.^[32]^

#### 2.1.1. *Type of participants*.

The population of interest is adults, more than 18 years old, with an explicitly diagnostic criterion of CRSwNP. In addition, patients with other diseases will be excluded, such as diabetes, allergic fungal rhinosinusitis (AFS), hemangioma, papilloma, cystic fibrosis, primary ciliary dyskinesia, peptic ulcer disease, myasthenia gravis, HIV or other immunodeficiency, uncontrolled systemic diseases, and so on. CRSwNP patients who younger than 18 years old or taking immunosuppressive medications for any reason were also excluded.

#### 2.1.2. *Interventions*.

According to the literature, interventions of corticosteroids for CRSwNP patients after ESS are of wide variation and can be divided into the following types: intranasal corticosteroids, oral corticosteroids, atomization/nebulization, nasal irrigation, direct infiltration, steroid-eluting stent, etc.

#### 2.1.3. *Comparators*.

There are a series of types of comparator conditions which will be eligible for inclusion in the network of evidence. The types of control conditions may include no another intervention of corticosteroid except intranasal corticosteroids, or pharmacological placebo, or 1 intervention of corticosteroid that is different with experimental groups.

#### 2.1.4. *Outcomes*.

The primary outcomes are efficacy (e.g., effective rate or cure rate), visual analogic scale of symptom severity, Lund-Kennedy endoscopic score. Certainty, adverse events/side effects will be record after interventions of corticosteroids.

#### 2.1.5. *Study designs*.

The systematic review will only include randomized controlled trials. These trials must have a sample size of at least 10 participants per condition. To reduce heterogeneity, crossover trials will be excluded. Finally, the language of included studies is restricted to Chinese and English.

### 2.2. *Information sources and search strategy*

We will perform a comprehensive electronic search of the medical literatures using medical subject heading (MeSH) and text related to management of CRSwNP after ESS. The search strategy was developed and reviewed by three independent reviewers (R ZHANG, H LIU, and P LI). We will search PubMed, Embase, the Cochrane Library, ClinicalTrials.gov, Web of Science, Chinese National Knowledge Infrastructure, Wangfang and VIP journal database. The retrieval time limit is from all databases establishment to July, 2022. The search strategy in PubMed was showed in Table S1, Supplemental Digital Content, http://links.lww.com/MD/H981. We will also complete the rest of the search based on specific requirements of other databases. There are no restrictions on publication date. The search will encompass all the indexed articles, computerized literature databases supplemented by manual searching of reference lists from each relevant paper identified.

### 2.3. Data collection and analysis

#### 2.3.1. *Study selection procedure*.

**Figure 1.**
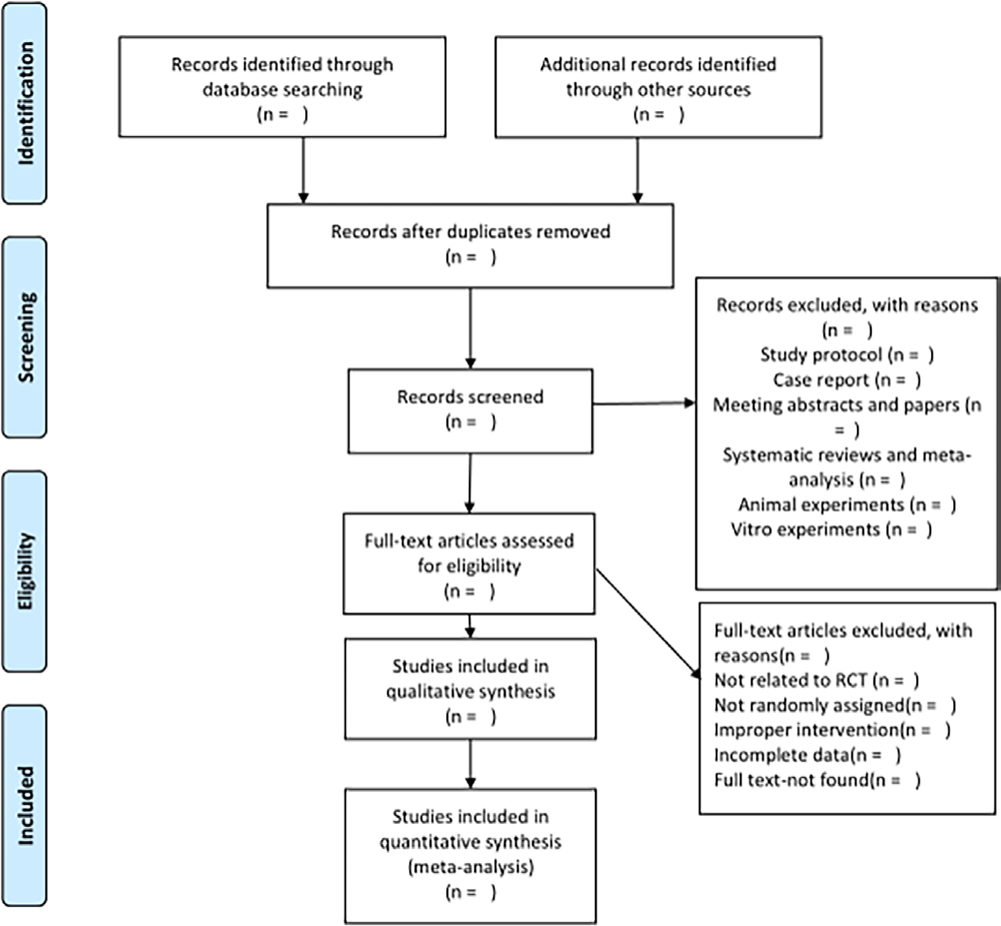
Flow diagram of the study selection process.

Detailed citations for the studies identified through the application of the search strategy will be exported to Endnote 20. Automatic and manual de-duplication will be carried out in Endnote 20. The abstracts found in multiple searches to identify potentially eligible articles will be retrieved and full-text articles reviewed to determine eligibility. Inconsistencies will be solved by discussion among independent investigators (J LIAO and H SHEN). Study authors will be contacted in the case of missing or incomplete data. Reasons for the discarded studies will be noted in detail. All data will be compiled in Micorsoft Excel Spreadsheet for analysis. The process and results of studies selection will be presented in a PRISMA diagram (Fig. 1).

#### 2.3.2. *Data extraction*.

The paper will be screened by 2 independent reviewers (J LIAO and H SHEN). Any disagreement will be resolved by consensus, and if possible, in discussion with a 3rd reviewer (L TIAN). For multi-arm trials, we will include all arms following the guidance of Dias et al.^[33]^ The data to be extracted includes authorship list, year of publication, countries of study, diagnostic criteria, group sample size, age distribution, gender distribution, intervention and comparator(s) characteristic, total study duration, outcomes as outlined in the PICOS. Source of funding will be collected. Follow-up length will be classified as short-term (≤3 months), medium-term (3 months–1 years), or long-term (>1 years) follow-up.

#### 2.3.3. *Classifications of arms*.

Classifications of arms will be carried out at the data extraction stage. The arms of each trial will be classified according to the type of intervention of corticosteroids, type of control condition employed, where applicable.

#### 2.3.4. *Risk of bias*.

For studies which are passed through the full-text screening stage, risk of bias will be assessed independently by 2 reviewers (J LIAO and H SHEN) using a revision of version 2 of the Cochrane risk of bias tool (RoB 2.0).^[34]^ We subclassified “some concerns” judgements as “some concerns probably high” and “some concerns probably low.” If at least 1 domain was high or probably high risk, we considered the study to be at high risk of bias.

#### 2.3.5. *Heterogeneity assessment*.

The different courses of intervention of corticosteroids may be treated separately in the outcome analysis, considering the subgroup analyses and/or meta-regression.

### 2.4. *Summary measures*

Summary measures will be reported for primary outcomes. For studies where continuous data is employed, the summary measures produced will be mean differences between treatment arms (with 95% confidence intervals). Any studies for which we cannot obtain 2 out of the three of baseline, change and follow-up (or allows us to compute this through confidence intervals or standard errors). We expect that these decisions will reduce bias while maintaining an informative evidence network. The data will also be summarized using treatment rankings and a surface under the cumulative ranking curve to aid the interpretation of the comparative effectiveness of all intervention types included in the network.

### 2.5. Data synthesis

A descriptive summary of pertinent study methodological and clinical characteristics will be initially reported. This will include summaries of key study and patient traits, included interventions, reported outcomes, and risk of bias assessments.

#### 2.5.1. *Pairwise meta-analysis*.

Meta-analysis using a random-effects model will be performed where studies are judged to be of adequate clinical, methodological, and statistical heterogeneity (*I^2^* < 50%).^[35]^ For study-level and pooled results, dichotomous data will be expressed as odds ratios (OR) with 95% confidence intervals and continuous outcomes will be presented as mean differences (MD) with 95% confidence intervals. Analysis will be carried out using Review Manager software (Version 5.3).

#### 2.5.2. *Network meta-analysis*.

To compare all interventions, network meta-analysis for each outcome is planned. Analyses will be performed using Stata software 16.0 and R software 4.2.0. If potential inconsistency is identified, we will explore the characteristics of the studies in the analysis and perform additional analyses to identify a remedy to resolve its presence. Pairwise comparison will be reported using the appropriate summary estimates with 95% credible intervals. Network geometry will be presented both with network graph, descriptively summarizing interventions. Results will be presented using forest plots and/or league tables, as well as summarized in the manuscript text. We will also present values of the Surface Under the Cumulative Ranking curve for each treatment as well as treatment rankings.^[36]^

#### 2.5.3. *Additional analyses*.

Exploratory analyses will be carried out, where there is a sufficient amount of information available to do so. These analyses will focus on the covariates the age distribution and gender distribution. Network meta-regressions will be conducted to individually examine the influence of these covariates on effect size estimates.

## 3. Discussion

### 3.1. *Contribution to literature*

Certainty, there are some drawbacks in some types of interventions of corticosteroids. According to many clinicians’ observations, the most common site of recurrence of nasal polyps was the frontal sinus region (around the frontal ostium and frontal recess, followed by frontal sinus cavity), owing to nasal sprays cannot always reach the middle meatus, frontal sinus, or frontal recess. While atomization/nebulization requires specific devices, which are not convenient for the patients to handle. Also, very small particles will overshoot the nasal passage and pass all the way through to the lower airway. As for nasal irrigation, the drug will always be diluted, and gravity will easily cause the drug to flow away soon. As we all know, oral corticosteroids will bring some systemic adverse events including osteoporosis, bone fracture, infections, and gastro-intestinal bleeding, and so on. Direct infiltration (including drugs drip into nasal packing materials or into nasal cavity) will achieve a local high-dose steroid treatment, also be safer than oral steroids. Steroids could deposit in the nasal cavity and the oropharynx by nasal dripping. While steroids deposited in the oropharynx could be swallowed and eventually absorbed from the gastrointestinal tract. Steroid-eluting sinus stents are inserted into the nose or paranasal sinuses to achieve local, controlled release of a known dose of corticosteroids drug directly into the sinus mucosa. However, cost-benefit constraints impair the wide clinical use of steroid-eluting sinus stents.^[37]^ Despite those interventions of corticosteroids have each advantage and disadvantage, this systematic review and network meta-analysis is to explore which 1 is the best corticosteroid of intervention for CRSwNP patients after ESS. Furthermore, the network meta-analysis will quantitatively compare different interventions of corticosteroids on CRSwNP patients after ESS in order to identifying the most efficacious 1. Eventually, the results of this study will provide evidence-based justification for choosing certain intervention of corticosteroids over others.

### 3.2. Limitations

Though the network meta-analysis methodology has become a critical component of evidence synthesis and decision-making in healthcare, there are potential drawbacks that may require consideration, such as the observational nature of indirect comparisons, assumptions that underlie the model and issue with inconsistency.^[38]^

### 3.3. Implications of this review

To our knowledge, no previous review has conducted a network meta-analysis comparing the efficacy and safety of different interventions of corticosteroids on CRSwNP patients after ESS. The current review will provide a clear direction for future research in the area of different interventions of corticosteroids on CRSwNP patients after ESS.

## Author contributions

**Conceptualization:** Lan Zhang, Baohua Zhu, Rong Zhang, Li Tian.

**Investigation:** Jie Liao, Hanchao Shen.

**Methodology:** Huixia Liu, Peishan Li.

**Supervision:** Baohua Zhu, Li Tian.

**Writing – original draft:** Lan Zhang, Baohua Zhu.

## Supplementary Material
