# Supplementary material for: The efficacy and safety of several interventions of corticosteroids for CRSwNP patients after endoscope sinus surgery: A protocol for systematic review and network meta-analysis
Source: Medicine (Baltimore). 2022 Nov 18;101(46):e31831. doi: 10.1097/MD.0000000000031831 (PMC9678636; doi:10.1097/MD.0000000000031831)
Supplement: Supplementary file 1 [file medi-101-e31831-s001.pdf]

Table 1 Search strategy in PubMed

Table 1 Search strategy in PubMed	
#1	"Steroids/therapy"[Mesh] OR “steroid” [Text Word] OR “corticosteroids” [Text Word]
#2	"sinusitis"[MeSH Terms] OR sinusitis [Text Word]
#3	"Nasal Polyps "[Mesh]
#4	#2 AND #3
#5	"endoscopy"[MeSH Terms] OR “endoscopic sinus surgery” [Text Word]
#6	"Nasal Surgical Procedures/therapy" [Mesh]
#7	#5 OR #6
#8	"Randomized Controlled Trial" [Publication Type]
#9	#1 AND (#2 AND #3) AND (#5 OR #6) AND #8
